# Physiological and genetic regulation of anhydrobiosis in yeast cells

**DOI:** 10.1007/s00203-023-03683-w

**Published:** 2023-10-02

**Authors:** Wioletta Sęk, Anna M. Kot, Alexander Rapoport, Marek Kieliszek

**Affiliations:** 1 Department of Food Biotechnology and Microbiology, Institute of Food Sciences, Warsaw University of Life Sciences, Nowoursynowska 159C, 02-776 Warsaw, Poland; 2 Laboratory of Cell Biology, Institute of Microbiology and Biotechnology, University of Latvia, Jelgavas Str., 1, Riga, 1004 Latvia

**Keywords:** Anhydrobiosis, Dehydration, Rehydration, Yeast

## Abstract

Anhydrobiosis is a state of living organisms during which their metabolism is reversibly delayed or suspended due to a high degree of dehydration. Yeast cells, which are widely used in the food industry, may be induced into this state. The degree of viability of yeast cells undergoing the drying process also depends on rehydration. In an attempt to explain the essence of the state of anhydrobiosis and clarify the mechanisms responsible for its course, scientists have described various cellular compounds and structures that are responsible for it. The structures discussed in this work include the cell wall and plasma membrane, vacuoles, mitochondria, and lysosomes, among others, while the most important compounds include trehalose, glycogen, glutathione, and lipid droplets. Various proteins (Stf2p; Sip18p; Hsp12p and Hsp70p) and genes (*STF2; Nsip18; TRX2; TPS1* and *TPS2*) are also responsible for the process of anhydrobiosis. Each factor has a specific function and is irreplaceable, detailed information is presented in this overview.

**Graphical abstract:**

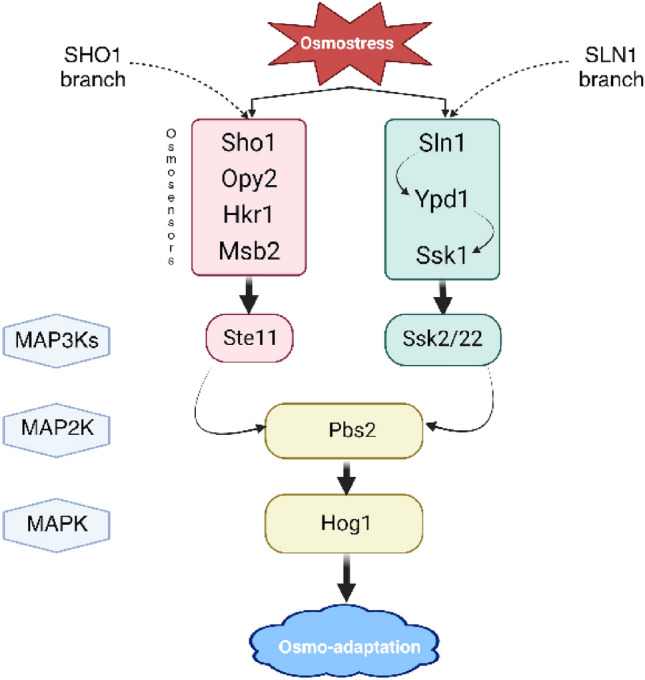

## Introduction

**Fig. 1:**
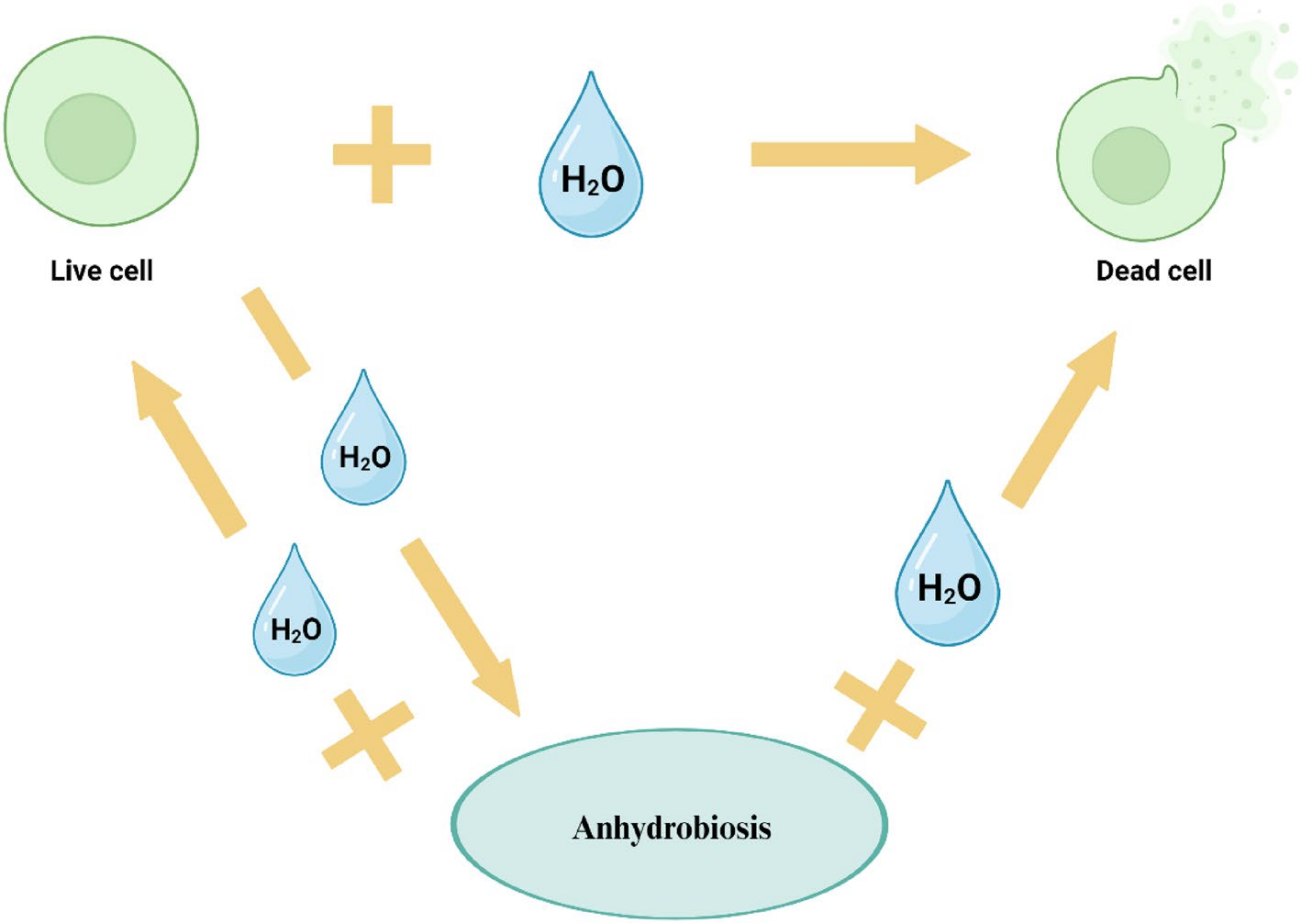
Diagram of the water cycle in the process of anhydrobiosis (Rapoport et al. 2019)

Very low temperatures during winter periods, or high temperatures during periods of drought, have contributed to the development of microorganism cells' ability to survive in extreme environmental conditions. The cold causes microorganisms to freeze, and their subsequent thawing and return to normal functioning are linked with the state of cryobiosis. When high temperatures cause microbial cells to lose water (dehydration) they may enter into the state of anhydrobiosis and then when climatic conditions change and cells reacquire a sufficient amount of water (rehydration) they may return to a normal active physiological state (Rapoport et al. 2019). Extreme and inadequate environmental conditions may lead to a temporary and reversible suspension of microbial metabolism—the state of anabiosis (or cryptobiosis). It is noteworthy that in some cases these processes can lead to irreversible cellular changes resulting in cell death (Fig. 1). Sometimes transition of living organisms into a state of anhydrobiosis is not possible. This process is directly affected by the presence of desiccation resistance systems and mechanisms; it depends on the initial state of the cells, or the presence of other stressors that, in combination with drought, will not allow the organism to survive. The reversible suspension of metabolic processes of yeast cells caused by lack of water in the cells (anhydrobiosis) is the subject of this literature review.

### Characteristics of the anhydrobiotic state

Anhydrobiosis is a state of living organisms during which their metabolism is suspended or delayed. It is a reversible process, but it may be associated with the loss of some amounts of valuable cellular components (Fig. 1) (Rapoport et al. 2014). A detailed scheme of the main events taking place during cell desiccation was presented in the review of (Dupont et al. 2014). It shows that the most important changes in the cells are linked with the period when water activity decreases to 0.5. There are reversible changes in the cytoskeleton, enzymes activities modifications/metabolic alterations, plasma membrane invaginations, beginning of phase transition of membrane lipids during this period (Dupont et al. 2014). A high degree of dehydration of yeast cells can be caused by both the industrial treatments used and unfavorable natural conditions (Rapoport et al. 2019). Under industrial conditions, the drying process can be carried out by various methods, and parameters can be adjusted to avoid undesirable physical, chemical, and biotechnological changes in the biological material. Drying methods used for industrial-scale yeast production include spray drying, freeze drying, and fluidized bed drying (Cioch-Skoneczny et al. 2018; Rapoport et al. 2016).

The degree of viability of yeast cells subjected to the drying process also depends on the rehydration of these microorganisms. The purpose of this process is to restore the microorganisms to the activity and properties they had before drying. Active dried yeasts that are used in the brewing industry have residual humidity of around 8%, and their rehydration stage is necessary before this yeast is added to the beer wort. The key parameter during rehydration is the temperature, which should be between 35 and 40 °C. After the rehydration process, a weight loss of up to 30% is noticeable, which is mainly due to the leakage of various substances from the microbial cells (Rodríguez-Porrata et al. 2008; Jenkins et al. 2011; Cioch-Skoneczny et al. 2018).

### Anhydrobiosis in yeast

Yeasts are one of the most important groups of microorganisms, used extensively in food technology in brewing, baking, winemaking, and distilling, among other applications. Their long-term storage must not adversely affect their biochemical and physiological properties, and, above all, those characteristics that are crucial for food production. While various active dry preparations of baker's yeast exhibit notably high quality, the viability of other dry yeast preparations, such as those used for wine and ethanol production, might be compromised once they are rehydrated and reactivated. This underscores the importance of enhancing our comprehension of anhydrobiosis—the state of extreme dehydration—and the factors that contribute to a successful transition of yeast into this state (Trofimova et al. 2010; Dauss et al. 2021). It has been observed that yeast cultivated in nutrient-rich media tends to yield more robust populations compared to cells grown in less nutritious synthetic media. Moreover, when it comes to bakers' yeast (*S. cerevisiae*), cells in the stationary phase exhibit higher resistance to the process of dehydration and subsequent rehydration, whereas the viability of cells in the exponential phase is considerably compromised by dehydration (Beker and Rapoport 1987). Through a gradual rehydration process using water vapor, it has been suggested that both magnesium and calcium play crucial roles in stabilizing yeast cell membranes. Notably, calcium ions have been found to enhance resistance to yeast cell dehydration, especially in cultures that are sensitive to stress during the exponential growth phase. These findings open up potential new avenues for augmenting the stability and viability of yeast during dehydration, which has implications, for instance, in the production of active dry baker’s and winemaking yeasts. Furthermore, it has been demonstrated that by carefully considering the availability of metal ions, it is possible to dehydrate exponential-phase cells of *S. cerevisiae* while maintaining high cell viabilities (Trofimova et al. 2010).

Environmental humidity and water activity are key factors responsible for yeast survival. Water participates in basic cellular functions and is responsible for the stability of cell membranes, DNA, RNA, and proteins. It is necessary for various biochemical reactions, such as enzymatic reactions and hydrolysis. The minimum value of water activity (a_w_) for most yeast strains is 0.80–0.88, although some osmophilic yeasts can survive even at a_w_ < 0.6. To survive in the event of complete water scarcity, yeasts have developed the ability to suspend and slow down their metabolism by entering a state called anhydrobiosis. They achieve this state when the intracellular water content reaches 8% to 10% (Câmara and Sant’Ana 2021).

### Characterization of the yeast cell wall-membrane complex

#### Cell wall

The yeast cell wall performs many important functions, being responsible for protection against biological, chemical, and physical agents, as well as against intracellular factors such as turgor pressure. Moreover, the cell wall selectively captures and excretes various compounds enabling intercellular communication, adhesion, and transmission of signals into the cell. It also enables the transfer of compounds into the cell and their excretion. It consists mainly of mannoproteins, chitin, and β-glucans. Studies have demonstrated that the composition of the wall can change under various stress factors. An example is Hsp12p proteins, compact hydrophilic proteins with characteristics resembling those of Late Embryogenic Abundant (LEA) proteins. Its concentration significantly rises after exposure to heat shock, during the transition into the stationary growth phase, and under conditions of diverse osmotic stress in yeast (Karreman et al. 2005). Hsp12p proteins can also be formed when the hydrostatic pressure is high (over 100 MPa) (Grajek and Szymanowska 2008). Through immunocytochemical assessments, it has been revealed that Hsp12p is predominantly situated within the cell wall, with a lesser amount surrounding the plasma membrane. Notably, yeast lacking the Hsp12p protein (Δhsp12 yeast) exhibited substantially reduced alterations in cell volume following osmotic shock (Motshwene et al. 2004). Based on these observations, it was hypothesized that the primary function of Hsp12p is to augment the flexibility of the yeast cell wall (Karreman et al. 2005, 2007).

Anhydrobiosis leads to the occurrence of significant changes in all components of the cell wall (Rapoport et al. 2019). The process of anhydrobiosis leads to damage of mannoprotein layers, resulting in the formation of densely cross-linked fibers with a radial orientation, which is similar in appearance to the fibers found in flocculent strains of brewer's yeast (Rapoport and Beker 1983; Rapoport et al. 2019). Moreover, the mannoprotein branching that occurs early in dehydration is associated with the process of yeast cell aggregation during rehydration, when large cell conglomerates are formed (Ventina et al. 1984). In addition to the changes that occur in the structure of the mannoprotein layer, during the rehydration of yeast cells, there is a reversible increase in the negative charge on the cell surface (Rapoport and Beker 1985; Rapoport et al. 2019). Dehydration of cells significantly affects their shape and size. After dehydration, wrinkles, depressions, and protrusions of various forms appear, which clearly indicate emerging conformational changes in cell wall components responsible for maintaining normal cell stiffness and shape. These changes can also be caused by damage to specific bonds occurring between glucans and mannoproteins. These changes are reversible after the rehydration of yeast cells (Rapoport et al. 1986a). It is also worth noting that dehydration does not affect quantitative changes in cell wall components and the carbon skeleton of yeast β-D-glucans (Borovikova et al. 2016). In studies of yeast cells, in addition to determining the size of dehydrated cells, it is also possible to determine the shape index, which is the ratio of cell length to cell width. A study showed that this ratio increased with the level of cell dehydration from 1.40 to 1.52, indicating that the cell elongates during the dehydration process (Rapoport et al. 1986a, 2019).

The yeast cell wall also contains proteins that affect the yeast's resistance to dehydration. They can be divided into three groups based on their mode of incorporation into the wall. The first group includes soluble cell wall proteins. They are non-covalently attached to the glucan matrix and mostly show homology with glycosylases. Proteins belonging to the second group are covalently bound to glucan via an ester bond between glutamines contained in a characteristic repeat sequence and glucose. The third group includes proteins anchored to β1,3-glucan by GPI and β-1,6-glucan anchors. These proteins are responsible for cell surface protection and cell wall synthesis (Borovikova et al. 2016; Rapoport et al. 2019). It was revealed in this study that mutants lacking proteins non-covalently attached to the cell wall and Pir proteins possess essentially lower resistance to drying and subsequent rehydration than the wildtype strain. A cluster of cell wall proteins that are chemically bonded together is referred to as the Pir proteins. These were initially named based on three genes that were assumed to encode proteins containing internal repetitions (Pir). Later on, they were recognized as cell wall proteins named Ccw1, Ccw2, Ccw3, and Ccw4p, all covalently linked. In the case of *S. cerevisiae*, there are five recognized PIR genes (PIR1/CCW6; PIR2/CCW7/HSP140; PIR3/CCW8; PIR4/CCW5/CIS3; PIR5), responsible for producing proteins with differing quantities of repeating segments, ranging from 1 to 10 units (Ecker et al. 2006).

Unexpectedly, the absence of the GPI-anchored cell wall protein Ccw12 led to the increase in yeast resistance to the same process of dehydration–rehydration. The authors suggested that this effect is linked to the compensatory synthesis of chitin (Borovikova et al. 2016). Thus, the structure and composition of the cell wall are among the most important factors strongly influencing the viability of yeast during the process of cell transition into the state of anhydrobiosis and subsequent restoration of its normal physiological activities.

#### Plasma membrane

Several changes occur in the structure of the plasma membrane in the presence of stress factors. During the process of cells’ transition into the state of anhydrobiosis, there are membrane folds that are necessary for the maintenance of membrane integrity in the conditions of a strong decrease in cell volume. Nevertheless, in these conditions there also occurs an increase in plasma membrane permeability (Rapoport and Kostrikina 1973; Biryuzova and Rapoport 1978). It is supposed that the increase in membrane permeability is linked with the changes in its molecular organization. In the proposed model in the conditions of cell dehydration, there is a phase transition of membrane lipids from the liquid crystalline phase to the gel phase. At the rehydration of dry cells, back phase transition takes place, and it may be very dangerous for the cells if this happens in the water medium. It may lead to high permeability of the membrane as well as the serious damage to its integrity. Fortunately, these negative effects may be prevented by reaching this back phase transition in waterless (for example, in water vapors) conditions before the beginning of the rehydration procedure or by rehydration at temperatures which are higher than the phase transition temperature (Crowe et al. 1989). A complementary hypothesis to this one supposes the partitioning of amphiphilic molecules into the membrane during drying. It may lead to local defects in membranes and promote leakage during rehydration (Golovina et al. 1998; Golovina and Hoekstra 2002; Hoekstra and Golovina 2002). According to Wharton (Wharton 2015), the process of drying may promote chromosome breakage, allowing foreign genetic material to be taken up and incorporated into the native one. This also suggests that the anhydrobiosis state may support gene-silencing techniques such as RNA interference. Such a technique can be facilitated by dehydration followed by rehydration in a solution containing double-stranded RNA. At the same time, it was found that one of the main intracellular protective reactions in the cells resistant to dehydration-rehydration procedures is chromatin condensation that takes place at the earlier stages of dehydration and protects DNA from damage (Rapoport and Kostrikina 1973). Dehydration leads to structural changes in the plasma membrane, i.e., a reduction in the distance between phospholipids and the ordering of hydrocarbon chains. In contrast, the disaccharide trehalose shows a protective effect during dehydration, as it interacts with the polar groups of phospholipids, increasing the distances between lipids and disrupting the structure of hydrocarbon chains (Crowe 2002). Ergosterol also plays an important role in maintaining the integrity of the plasma membrane structure during cell dehydration (Dupont et al. 2011). Moreover, damage to membrane proteins can adversely affect cell viability during dehydration and rehydration (Rapoport et al. 2019). Also worth mentioning are aquaporins, which are integral membrane proteins that form channels that participate in the process of water transport, as well as gene expression. Another group of proteins that may be produced during drying are heat shock proteins. This type of protein can bind to proteins denatured during drying, thereby preventing their aggregation. Heat shock proteins also play an important role in the rehydration process, when they participate in the repair of damaged proteins and membranes during water recovery (Guzhova et al. 2008; Wharton 2015).

## Limitations of the mechanical effects of anhydrobiosis on the mechanical structure of yeast cells

It has been found that there are large differences between the survival rates of yeast strains during their transition into the state of anhydrobiosis. Halotolerant, thermo-resistant, and psychrophilic yeasts are more resistant to dehydration than mesophilic ones (Khroustalyova et al. 2001; Rapoport et al. 2014; Khroustalyova and Rapoport 2019). In addition, the mortality of the cells during their dehydration can be decreased by incubating cells in the solutions with increased osmotic pressure (Rapoport and Beker 1983). To date, the mechanisms of cell transition into anhydrobiosis still are not fully understood, so it is important to determine them and the main factors that allow cells to maintain their viability during dehydration and rehydration. It has also been shown that during the processes of dehydration and rehydration all intracellular organelles undergo distinct structural changes (Rapoport et al. 2016, 2019).

Research on the reactions occurring inside cells as a result of their dehydration conducted at the turn of the twentieth century has provided a lot of important information. It has been proven that the transition of yeast into a dormant state is associated with changes in the shape and size of its cells and cell wall structures. The change in shape is influenced by vacuoles, which decrease in volume during dehydration. Invagination reactions are observed in the cytoplasmic membrane in the dehydration state. This is a biological morphogenetic process that leads to invagination of the upper layer, and in the case of dehydration, this means invagination in the membrane. One can also see an increase in lysosome activity in yeast cells both during dehydration and subsequent rehydration. The shape of the cell nucleus and its membrane is altered, which affects cell survival after anhydrobiosis. According to these studies, the most stable organelles during dehydration and rehydration are the mitochondria (Rapoport et al. 2019).

The mitochondria are an important organelle, mainly responsible for the production of adenosine-5′-triphosphate (ATP) during cellular respiration and for regulating cell metabolism. For the normal physiology of yeast cells, it is important to maintain proper mitochondrial lipid metabolism. Mitochondria are home to phosphatidylserine decarboxylase, associated with the synthesis of phosphatidylethanolamine, as well as mitochondrial sphingolipids, responsible for the integrity, polarity, composition, and function of cytochrome c oxidase (Malina et al. 2018). Therefore, it is clear that one of the main factors ensuring cell viability during their transition into the state of anhydrobiosis must be related to maintaining mitochondrial stability (Hagen et al. 2004). Damage to yeast cells during dehydration and rehydration can be related also to oxidative stress, and therefore to reactive oxygen species (ROS), which are produced as by-products of ATP production. It is known that ROS have a dual role in cells’ activities – they may have constructive and destructive functions. It was shown that ROS function as signaling molecules and control several normal physiological functions. At the same time ROS are linked with various pathological states (Bardaweel et al. 2018) that can damage various macromolecules and lead in extreme cases to cell death. In the case of yeast cells transition into the state of anhydrobiosis ROS are supposed as one of the most dangerous factors at both stages—at dehydration and rehydration of the organisms. Dehydration of cells is connected with the formation of ROS by mechanisms related to metabolic arrest and enzyme dysfunction (Garre et al. 2010). Fortunately, there are a number of intracellular protective compounds including catalase and superoxide dismutase that help yeasts to maintain viability in these conditions. Over the past decades, increasing attention has been paid to information about the antioxidant properties of ergosterol. It became clear that it is one of the most efficient compounds that provides both structural functions of the yeast plasma membrane and protects it from oxidation (Dupont et al. 2012). Ergosterol accumulated in the plasma membrane of yeast cells may be responsible for the protection of phospholipids against oxidative perturbations. It is supposed that the presence of ergosterol close to the unsaturated phospholipid acyl chains of yeast is very important for the cells and explains their resistance to oxidative stress during desiccation and following rehydration/reactivation. There is a hypothesis that the oxidation of membrane ergosterol may be a protective reaction that avoids the peroxidation of phospholipids (Dupont et al. 2021). Some other intracellular protective compounds such as trehalose, glycerol, and glutathione that also have antioxidant properties will be discussed below. All this information means that mitochondria, in addition to the obviously important protective role they play in maintaining cell viability, such as under stressful conditions, in some cases can also be a source of compounds that negatively affect cell function (Bouchez and Devin 2019). Moreover, mitochondrial DNA (mtDNA) is of particular importance in the process of the transition into anhydrobiosis, as it is highly sensitive to harmful external factors, but shows high stability during dehydration and rehydration. Moreover, the state of mitochondria is important for the proper viability of yeast cells during dehydration, and the resistance of mtDNA to anhydrobiosis affects yeast cell survival (Jenkins et al. 2010). In addition, of all cellular organelles, mitochondria show the least extensive structural changes during dehydration and rehydration and recover earlier. The most important change in mitochondria has been linked to mtDNA condensation, which has a protective function for the mitochondrial genome. These mechanisms promote the production of sufficient energy, which is essential for the function of many intracellular repair processes occurring during the transition into anhydrobiosis and subsequent reactivation from this state (Rapoport and Kostrikina 1973). It also appears that the high structural stability of mitochondria may be related to the presence of two membranes surrounding these organelles (Rapoport et al. 2019). Also, the addition of lithocholic acid (LCA) has been linked to the protective function of mitochondria. Exogenously supplied LCA reduces mitochondrial fragmentation, increases resistance to oxidative and thermal stress, inhibits apoptosis, and increases the stability of nuclear DNA and mtDNA. The mechanism of action of exogenously added LCA involves the molecule entering yeast cells, which is then transported into the mitochondria, to the inner mitochondrial membrane, but also fuses with the outer membrane. Subsequently, under the influence of LCA, there are changes in the synthesis and movement of glycerophospholipids in both mitochondrial membranes, mitochondrial membrane lipids and in the size, number, and morphology of mitochondria, which, as a result, affect the functions of the organelles, i.e., membrane potential, the level of ATP synthesis and reactive transcription factor (RFT) homeostasis, as well as altering the mitochondrial proteome and its functionality (Khroustalyova and Rapoport 2019).

Vacuoles in yeast cells are organelles that are linked with many important functions such as degradation of molecules, storage of ions, and maintenance of the appropriate pH of the cell and are also associated with autophagy. The number and size of vacuoles depend on the conditions in which the yeast is cultured and the physiology of the cells themselves (Armstrong 2010). Since vacuoles contain a large amount of water, it is obvious that during dehydration there should take place a change in their size and shape. During the dehydration of yeast cells, large vacuoles are reduced and fragmented, and the number of small vacuoles changes (Rapoport et al. 1986a, b). Since vacuoles contain more concentrated solutions than other cell organelles, dehydration results in an increase in osmotic pressure due to an increase in salt concentration in the vacuoles. Then, under the influence of anhydrobiosis, the redistribution of proteins in certain areas of vacuolar membranes is altered, and the hydrophobic interactions of lipids with proteins are disrupted. As a result, these changes cause an increase in the permeability of vacuole membranes, for example, to magnesium and potassium ions, and the vacuole itself increases its lysosomal and phagosomal activity (Beker and Rapoport 1987).

In summary, during the transition into anhydrobiosis and subsequent reactivation from this state, many different structural changes occur in the organelles of yeast cells. The size of the cells decreases and the shape of the cells elongates, condensation of mtDNA occurs in the mitochondria, folds of the plasma membrane appear, and large vacuoles change shape and may begin to resemble the smaller ones. In addition, there is a condensation of chromatin in the nucleus, autolysis of those parts of the nucleus that do not contain chromatin, and structural changes of lipids in nuclear membranes. Also in the membranes of peroxisomes there are changes in the structure of the lipid layer (Rapoport et al. 2019; Khroustalyova and Rapoport 2019).

## Genetics of dehydration and rehydration in yeast cells

The functions of certain genes that may be linked to the tolerance of yeast cells to dehydration were studied (Rapoport et al. 2019). Special attention was attributed to STF2 and SIP18 genes, which encode hydrophilic-like proteins that regulate RFT production and apoptosis (Rodríguez-Porrata et al. 2012; López-Martínez et al. 2012). Hydrophilins (e.g., Stf2p) are antioxidants and stabilize membranes and proteins during dehydration–rehydration induced cellular stress (Novo et al. 2007). The Sip18p protein, located in the cytoplasm, has antioxidant capacity and is transported to the cell nucleus during dehydration (Vaudano et al. 2010). Other genes related to maintaining cell viability in dehydration include TRX2 (protection against oxidative stress), GPD1 (related to osmotic stress, responsible for glycerol biosynthesis), SSA3 and SSA4 (determination of expression of heat shock proteins belonging to the Hsp70 family), CTT1 (protection against oxidative stress), and HSP12 (Hsp12 protein synthesis). In addition, TPS1 and TPS2 genes are responsible for the synthesis of trehalose, which is important for the dehydration tolerance of yeast cells (Zambuto et al. 2017).

The transition into anhydrobiosis is also linked to changes in the genome at the transcriptional and translational levels. Yeast cells respond rapidly and efficiently to the dehydration process, implementing mechanisms that already at its early stages lead to the accumulation of intracellular glycerol, which acts as an osmoprotectant. The GDP1 gene, whose expression is under the control of the MAP-HOG (mitogen-activated protein- high osmolarity glycerol) signaling pathway, is associated with this mechanism. There is also the overexpression of GPP1 and GPP2 genes, which encode glycerol-3-phosphatases, as well as overexpression of the STL1 gene, which encodes the glycerol transporter Stl1 (Noti et al. 2015).

The rehydration process is also related to the activation of some genes of the fermentation pathway and the non-oxidative phase of the pentose-phosphate pathway, as well as genes related to ribosome biogenesis and protein synthesis (Novo et al. 2007). There is a resurgence of protein synthesis, and genes responsible for the synthesis of high-affinity (HXT2 and HXT4) and low-affinity (HXT3 and HXT1) hexose transporters, and nitrogen transporters adapt to hydration conditions, depending on the presence of glucose. The response of transporter genes to rehydration varies depending on their substrate affinity (Vaudano et al. 2010) [Vaudano et al. 2010]. Moreover, the MEP1, MEP2, and MEP3 genes, which encode ammonium transporters, are also involved in the rehydration stage and are not linked to osmoregulation (Vaudano et al. 2009).

During dehydration and rehydration of yeast cells, there are no structural changes in the karyotype, i.e., the size and number of chromosomes, nor changes in the DNA sequence, suggesting that transposons do not move during the transition into anhydrobiosis (Jenkins et al. 2011). During the transition into anhydrobiosis, there is significant degradation of total RNA, but cell viability is not affected, as it is mainly ribosomal RNA. Therefore, the nucleosome, which is responsible for ribosome biogenesis, is restored during rehydration and does not undergo significant damage or changes during the transition into anhydrobiosis (Taddei and Gasser 2012).

Mitochondria serve as the sites where oxidative phosphorylation occurs. The indispensability of an intact respiratory chain for sustaining life is beyond question. In the context of yeast cell physiology, mitochondrial lipid metabolism holds significant importance. Therefore, it's evident that a critical factor in ensuring cell viability is linked to upholding mitochondrial stability (Rapoport et al. 2019).

It is recognized that damage incurred by yeast cells during the process of dehydration and subsequent rehydration can also be attributed to oxidative stress (Dupont et al. 2014). This stress is closely associated with reactive oxygen species (ROS), which are generated as by-products of ATP production during oxidative phosphorylation within the respiratory chain. Consequently, mitochondria emerge as a primary source of ROS. Despite the notion that ROS are normal by-products of cellular metabolic processes, they have the potential to harm various macromolecules and, in adverse cases, trigger cell death (Kaeberlein 2010). This implies that while mitochondria play a crucial protective role in maintaining cell viability under stressful conditions, they can, in certain scenarios, also act as sources of detrimental compounds (Rapoport et al. 2019).

Therefore, it is imperative to meticulously investigate the functional activity of mitochondria during the phases of dehydration and rehydration, as well as any possible alterations they might undergo under these circumstances. Mitochondrial DNA is known to exhibit sensitivity to stress-induced treatments. In some instances, this can lead to the development of petite mutants. However, examinations of brewing yeast have not revealed an increase in the occurrence of petite mutants following dehydration treatment. Furthermore, various attributes of mitochondrial DNA, such as copy number, sequence rearrangements, and resistance to mutagenic challenges, remain unaltered after the dehydration of different strains of brewing yeast (Jenkins et al. 2010). Consequently, based on these findings, it can be inferred that mitochondrial DNA displays a relatively high level of stability under conditions involving dehydration and rehydration treatments (Rapoport et al. 2019).

In addition, ribosomes indirectly affect yeast cell viability during dehydration, as the 60S subunit competes for the binding of chaperone proteins that protect the proteome from dehydration. Thus, the reduced amount of ribosomal RNA increases the available pool of chaperone proteins, which in effect reduces the deleterious effects of dehydration on yeast cells (Welch et al. 2013).

Thus, immediately at the beginning of the rehydration phase, protein synthesis begins and overexpression of the mentioned genes occurs. In conclusion, the transition into the state of anhydrobiosis does not lead to serious damage to the genome of yeast cells. Most likely, this stability is related to specific intracellular reactions that provide the necessary protection of important structures and macromolecules.

## Reserve substances in yeast cells important for anhydrobiosis

### Trehalose

**Fig. 2:**
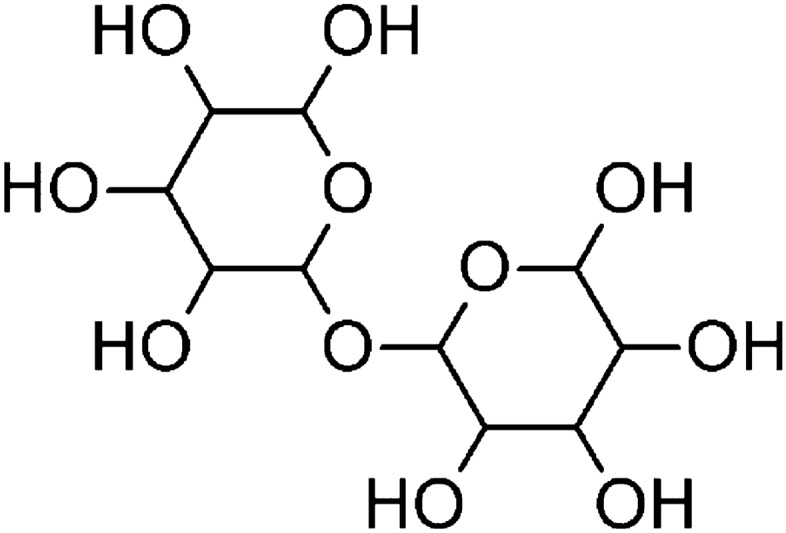
Structural formula of trehalose

Under various stress conditions caused by temperature changes, osmotic stress, dehydration, or cell contact with ethanol, yeast cells begin to accumulate large amounts of trehalose, a natural sugar composed of two glucose molecules (Fig. 2). The production of trehalose increases at 30 °C and continues to rise with higher temperatures. Studies have shown that the amounts of this disaccharide vary depending on the yeast strain studied. Thus, the ability to produce trehalose can be used as a factor in selecting stress-resistant strains for industry (Marino et al. 1989; Grajek and Szymanowska 2008). Trehalose provides carbohydrate reserves in cells and is a source of energy during starvation, which can be observed during the stationary phase of growth. The typical pathway for trehalose production involves two enzymes: trehalose-6-phosphate synthase (TPS), which facilitates the synthesis of trehalose-6-phosphate (T6P) from glucose-6-phosphate (G6P) and UDP glucose (UDPG), and trehalose-6-phosphate phosphatase (TPP), which removes the phosphate group from T6P to yield trehalose (Cabib and Leloir 1958; Kaasen et al. 1992; Lunn et al. 2014).

During stress conditions, trehalose has a protective function for proteins by stabilizing them through hydrogen bonds. It prevents denaturation processes and membrane fusion in the yeast cell. It is also responsible for membrane sealing (preventing electrolyte loss), and protects lipids from oxidation. The rapid increase in trehalose induces an increase in cell osmolarity, which results in an increase in the mechanical strength of the cell wall and its resistance to lytic enzymes (Grajek and Szymanowska 2008; Piecuch and Obła̧k 2013). At the end of the twentieth century, when the topic of anhydrobiosis started to attract significant attention, scientists from Italy (Marino et al. 1989) studied the behavior of trehalose and trehalase in yeast cells during the state of anhydrobiosis. Baker's yeast was the subject of the study. They observed a sharp increase in the level of trehalose after drying the cells, with simultaneous inhibition of trehalase activity and a decrease in glycogen content. After rapid rehydration for 15 min, the trehalose content decreased, while the amount of glycogen decreased further. The activity of trehalose synthase was stopped, while an increase in trehalase activity was noted. After a slow rehydration lasting 12 h, an even greater decrease in trehalose and glycogen content was observed, while enzyme activity was altered. After slow rehydration, inhibition of trehalase was observed while trehalose synthase was activated. As can be seen, after the cells returned to a functional state, trehalose was used for metabolic reactions; hence its content decreased. The decreasing content of trehalose, after rapid rehydration, was accompanied by an increase in trehalose degrading enzyme. Carrying out prolonged rehydration led to a state of cells that allowed trehalase to return to decreasing activity with an increased demand for trehalose synthase. Such a phenomenon could likely occur when the crisis condition was brought under control (after slow rehydration) and the yeast cells returned to normal functioning, while the authors of the described experiments did not explain the reason for such impressive changes in enzyme activity in dried and rehydrated cells (Marino et al. 1989).

An explanation for the causes of the phenomena described above was given in the review of Rapoport et al. (Rapoport et al. 2019). The researchers described the principle of water substitution to prevent imbibition leakage (imbibition is the process of swelling of colloids after the rehydration process). Extensive research by the team of Crowe (Crowe et al. 2001; Crowe 2002) has provided information that organisms that can accumulate high levels of trehalose or sucrose are more resistant to dehydration. In yeast, trehalose can replace the water in forming hydrogen bonds with the phosphate group of lipids. This phenomenon keeps the core of the membrane in a liquid crystalline state by providing the necessary distance between lipids in the membrane in the desiccated state (Rapoport et al. 2019). During rehydration, water molecules replace trehalose back into the membrane, ensuring that there is no phase transition in the membrane.

Additional information regarding trehalose as an important factor responsible for the survival of yeast cells under anhydrobiosis conditions is provided by the work of Wharton (Wharton 2015), who observed that in organisms capable of reversible changes during desiccation, there is an increase in the expression of a gene related to trehalose synthesis.

### Glycerol

**Fig. 3:**
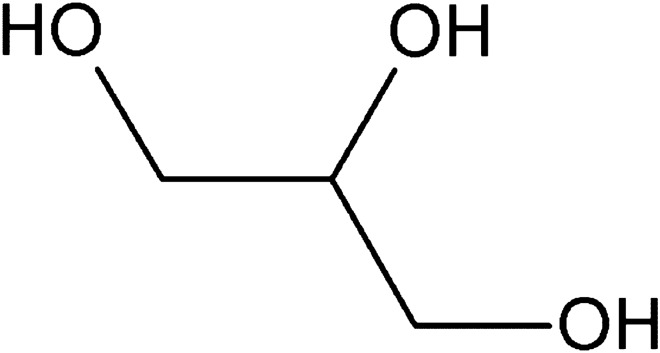
Structural formula of glycerol

**Fig. 4:**
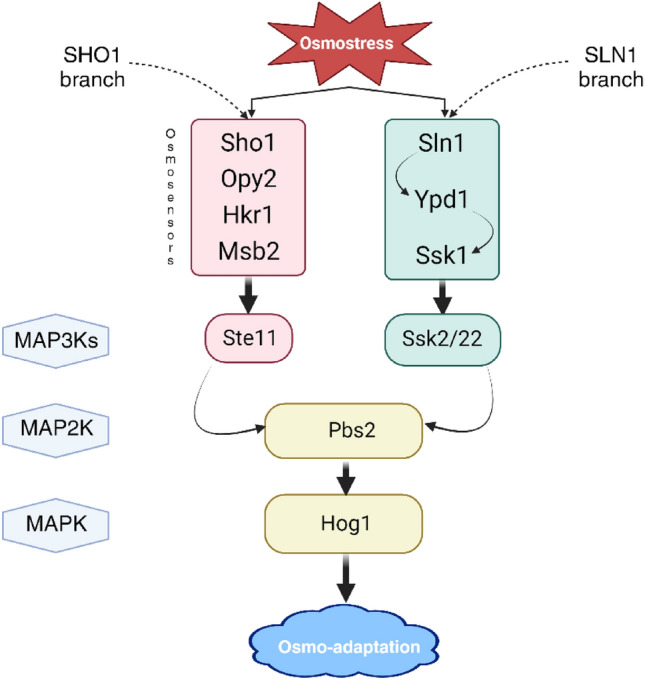
Schematic diagram of the HOG pathway (Tatebayashi and Saito 2023)

While trehalose is mainly synthesized as a result of different stresses, glycerol is produced mainly under increased osmotic stress (Grajek and Szymanowska 2008), which also takes place during the dehydration of yeasts. Glycerol is a triol, or the simplest trihydroxy alcohol (Fig. 3). It is also an important osmoprotective molecule in most yeast species. It can also serve as a carbon source, and the cytosolic level of its degradation/synthesis is an important factor in maintaining cellular redox balance (Duskova et al. 2015). It is also used for the synthesis of glycerophospholipids and triacylglycerols and serves to protect against oxidative or heat stress (Rapoport et al. 2019). To maintain optimal glycerol levels under changing external conditions and available nutrients, yeast cells tightly regulate glycerol synthesis, degradation, and transport. At high external osmotic pressure, when intracellular pressure needs to be increased, the high osmolarity glycerol (HOG) pathway signals cells to increase glycerol synthesis, take it up from the environment and reduce its loss from cells (Wang 2016; Rapoport et al. 2019). The HOG pathway comprises a central structure consisting of three tiers of protein kinases known as MAPK, MAPK kinase (MAPKK, MAP2K), and MAPKK kinase (MAPKKK, MAP3K). Furthermore, the initial segment of the HOG pathway includes two branches, SHO1 and SLN1, which have redundant functions but operate through distinct mechanisms (Fig. 4). When yeast cells encounter elevated extracellular osmolarity, the osmosensors within the SHO1 and SLN1 branches independently perceive the osmotic stress, leading to the activation of corresponding MAP3Ks. In the SHO1 branch, complexes formed by Sho1, Opy2, Hkr1, and Msb2 trigger the activation of the MAP3K Ste11. Meanwhile, in the SLN1 branch, the Sln1–Ypd1–Ssk1 phospho-relay system activates two functionally overlapping MAP3Ks, Ssk2 and Ssk22 (Ssk2/22). Following activation, both Ste11 and Ssk2/22 phosphorylate and activate the MAP2K Pbs2. Subsequently, activated Pbs2 phosphorylates the MAPK Hog1 at T174 and Y176 within its activation loop, thereby initiating its activation process (Tatebayashi and Saito 2023).

Hog1p is the last kinase of this pathway that is crucial for both the immediate and late response of cells to hyperosmotic stress, as it activates or deactivates various transporters and enzymes or induces necessary transcriptional and translational changes, such as in glycerol and sugar metabolism or in the cell cycle. Under osmotic stress, glycerol is massively synthesized de novo from dihydroxyacetone-P via glycerol-3-P in a Hog1-dependent manner (Duskova et al. 2015). Glycerol can readily permeate lipid membranes and is thus removed from cells by passive diffusion according to the concentration gradient. In yeast cells, on the other hand, there is a transporter, St1lp, that can actively accumulate glycerol from the environment across the plasma membrane using ATPase. Another transporter (exporter), Fps1p, which mediates the rapid release of glycerol from the cytosol by facilitated diffusion, is mainly activated when cells need to rapidly decrease intracellular turgor and increase intracellular water activity. Fps1p is a member of the major intrinsic protein family and facilitates the diffusion of other small solutes across the plasma membrane in addition to glycerol. A hyperosmotic environment causes Fps1p channel closure (mediated by Hog1p), while a decrease in external osmolarity causes this channel to open. Moreover, the activity of membrane proteins associated with glycerol transport – Stl1p (importer) and Fps1p (exporter) – is very important for the cell's ability to survive during its transition to anhydrobiosis (Duskova et al. 2015; Wang 2016; Rapoport et al. 2019).

The available scientific literature provides scant reports on the importance of glycerol as a protective agent for living organisms during osmotic dehydration. Interestingly and fundamentally, one of them is a study by Qiu and Bedding (Qiu and Bedding 2002) on the subject in the cells of the nematode *Steinernema carpocapsae*. Studies from 1997 to 2000 report that by using glycerol and trehalose as protective agents, the nematodes can survive drought conditions (Qiu and Bedding 1999, 2000). The mentioned protective agents replace free and structural water lost during dehydration. Thus, the cell maintains the structural and functional integrity of the cytoplasmic membrane. The function and importance of trehalose in nematode survival are well known, while the function of glycerol has been studied in a considerably smaller amount of research (Qiu and Bedding 2000). In the study described here, dehydration was performed using hypertonic solutions at temperatures of 5 and 23 °C. The study showed that low temperatures induce the accumulation of trehalose, but not glycerol – hence the conclusion that trehalose can act as a cryoprotectant. Swedish researchers reported that salt stress leads to an increase in the intracellular amounts of glycerol in the yeast *S. cerevisiae*. This compound prevents the loss of turgor pressure in the cell (Blomberg and Adler 1992). According to the researchers, glycerol plays a key role in balancing internal osmotic pressure, as glycerol synthesis is much more efficient than trehalose concerning osmotic balance in the cell. This is confirmed by the results presented by Qiu and Bedding, which showed that nematodes containing 1% glycerol (calculating for the dry weight) have the same protective effect as with 4% trehalose under the same conditions. An important argument supporting the essential role of glycerol in the dehydration stress response is the demonstration that initially the nematodes produced more glycerol than trehalose to quickly achieve osmotic balance. At a later stage, once equilibrium is achieved, the cells convert glycerol to trehalose, and this process occurs during dehydration. The research has provided important information on the protective agents of cells and has shown that each compound has its own specific functions and is irreplaceable.

### Lipid droplets

Lipid droplets (LDs) are lipid storage organelles derived from the endoplasmic reticulum (ER). LDs consist of a highly hydrophobic, non-polar core made up of lipids, surrounded by a monolayer of polar phospholipids, which also contain proteins that are on the surface of the LD or embedded in it and are involved in lipid metabolism and its regulation. The lipid droplet monolayer consists mainly of phosphatidylcholine, phosphatidylinositol, and phosphatidylethanolamine. The main storage lipids in yeast cells are triacylglycerols and sterol esters. Lipids in LD can be used by the cell, such as for energy production in β-oxidation and as material for intracellular membrane synthesis (Ventina et al. 1986; Wang 2016). During dehydration, lipid droplets are observed associated with the normal, disorganized, or circular ER membrane, suggesting that the ER membrane may be degraded and free fatty acids are stored in lipid droplets. Lipids can be consumed during dehydration to provide energy and be taken up in the early phase of dehydration by vacuoles, which degrade lipid droplets in a process resembling microautophagy (lipophagy) (Klug and Daum 2014). In addition, there is a positive correlation between the tolerance of yeast cells to anhydrobiosis, and the sum of sterol esters and triacylglycerols stored by these cells, as these lipids can contribute to various metabolic and repair processes that occur in yeast cells during their rehydration. Moreover, a positive correlation between yeast survival rate and a high percentage of unsaturated lipids has also been observed, which may provide adequate membrane fluidity in yeast cells during dehydration (Ren et al. 2020). The surface of lipid droplets can create an environment for the storage or inactivation of misfolded or aggregated proteins, which can occur during various stress treatments, including dehydration and rehydration processes, and thus they can co-create an important mechanism for the removal of proteins that form toxic aggregates.

### Glycogen

**Fig. 5:**
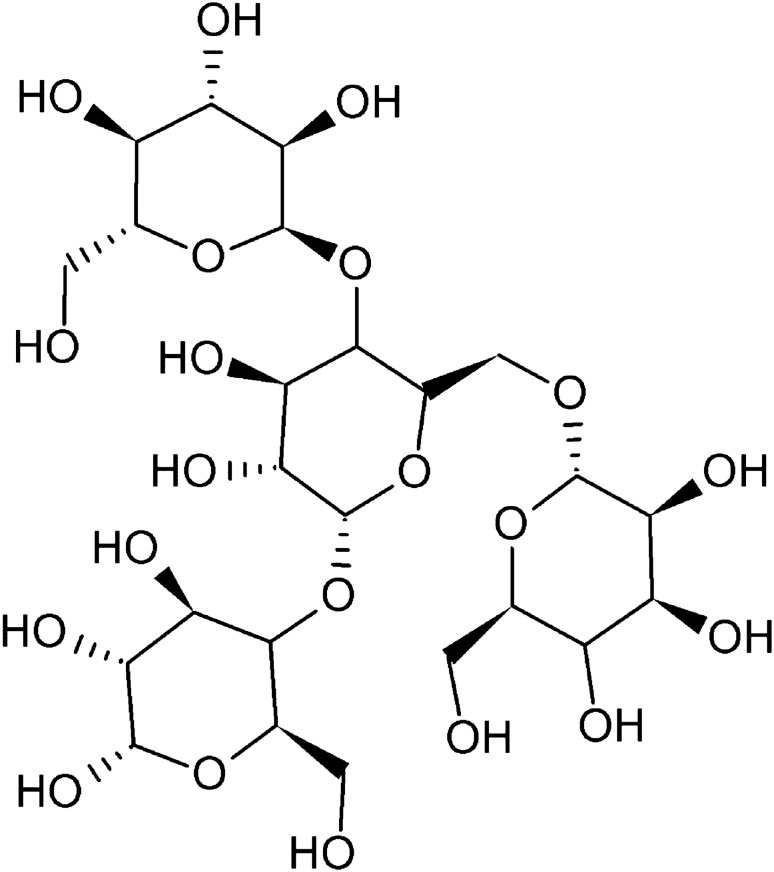
Structural formula of glycogen

Glycogen is a polysaccharide whose molecules are made up of linked D-glucose residues (Fig. 5). Glycogen is, along with trehalose, the main form in which glucose is stored in yeast cells. There are large differences in the content of these two compounds in cells in response to different environmental changes, indicating that their metabolism is controlled by complex regulatory systems, mainly cyclic adenosine-3′,5′-monophosphate (cAMP) and the protein kinases Snf1 and Pho8. The presence of glycogen and trehalose in the cell is beneficial for survival, as these compounds play significant roles in controlling glycolytic flux, stress response, and energy storage (François and Parrou 2001). The role of glycogen during anhydrobiosis is related to trehalose accumulation during dehydration, as with trehalose storage, there is significant activation of trehalose synthase and a sharp decrease in trehalase activity, leading to a significant decrease in glycogen content. This mechanism is related to the conversion of glycogen to glucose-1-phosphate (G-1-P), via glycogen phosphorylase, which in turn generates uridine diphosphate glucose (UDP-glucose), a substrate for trehalose synthase. Thus, reduced cellular glycogen content and increased UDPG levels can activate trehalose synthase and inhibit trehalase during yeast cell dehydration (Marino et al. 1989).

### Glutathione

**Fig. 6:**
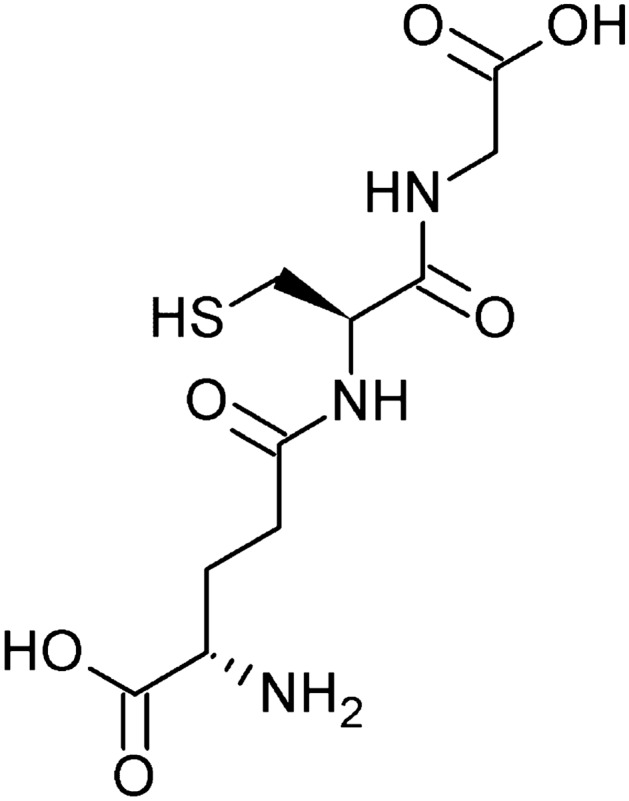
Structural formula of glutathione

Glutathione is a thiol tripeptide made up of three amino acids – glutamic acid, cysteine, and glycine – and is therefore also called L-γ-glutamyl-L-cysteinyl-glycine (Fig. 6). Glutathione is a very potent antioxidant; in eukaryotic cells, it is responsible for gene expression, iron homeostasis, or redox signaling. Its content in the dry cell substance of the yeast *Saccharomyces cerevisiae* is about 1% (Grajek and Szymanowska 2008; Kurylenko et al. 2019). Glutathione deficiencies can lead to many serious disorders in both humans and animals. Deficiencies of this compound result in neurological disorders, diseases such as HIV or diabetes, and even cancer. The compound is used in the food, pharmaceutical, and cosmetic industries. Glutathione has many applications in medicine and biotechnology and is used in delaying aging and treating liver disease and various eye and skin diseases. It is also used as a dietary supplement, and in food production, it is used to make cakes and stabilize the taste of low-alcohol products such as wine and beer. Because of such wide-ranging applications, glutathione production is growing every year. Up to 200 tons of the compound are produced annually worldwide (Kurylenko et al. 2019).

Currently, the main microbial producer of glutathione is yeast. In addition to the search for new efficient strains and optimization of the production system, genetic engineering is one of the main pathways for obtaining large amounts of microbial glutathione (Schmacht et al. 2017). For example, Kurylenko et al. (Kurylenko et al. 2019) subjected a new yeast strain obtained by genetic engineering – *Hansenula polymorpha* mcGSH2/MET4 (pGAP) – to anhydrobiosis. The possibility of producing active dry preparations of this strain would facilitate its implementation on an industrial scale and subsequent use. For this reason, the study involved dehydration and rehydration of the modified strain and the parent strain. The strain with an embedded gene conditioning high glutathione production showed very good, even ideal survival results after rehydration (close to 100%). Such a result is rare for genetically modified organisms. An important point was to check whether the new strain retains its production capabilities after rehydration. The results showed higher amounts of glutathione produced by the new *H. polymorpha* strain compared to the parental strain. Cells of the recombinant strain had a higher survival rate in both fast and slow rehydration methods. The new yeast strain proved to be more stable during dry storage than the parental strain, which can be explained by better protection of intracellular membranes during rehydration by larger amounts of glutathione produced. The process of anhydrobiosis did not affect the characteristics (production of increased amounts of glutathione) of the new yeast strain, which will allow the use of dry preparations in biotechnological processes on an industrial scale (Kurylenko et al. 2019).

### Autophagy in the process of anhydrobiosis

The word "autophagy" comes from the Greek *auto* meaning "self" and *phagos* meaning "eating." In other words, the whole word means "eating oneself." Autophagy is an evolutionarily conserved biological process that involves the removal of unnecessary or abnormal components from a cell. The phenomenon consists of four phases:

I. Induction involves the formation of an insulating membrane called a phagophore;

II. Phagophore elongation and conversion, fusion, to form a closed autophagosome with a double membrane, containing part of the cytoplasm and/or organelles;

III. The outer membrane of autophagosomes with the formation of lysosomes and autophagolysosomes;

IV. Degradation of the inner membrane of the autophagolysosome and its contents by lysosomal enzymes (Doherty and Baehrecke 2018).

As early as the 1960s, scientists first observed that cells can destroy their own components, including damaged proteins and entire organelles such as mitochondria. This unwanted waste is wrapped in membranes, forming sac-like vesicles (autophagosomes), and transported through "waste processing factories" called lysosomes. The autophagosomes fuse with the lysosomes, and their contents are broken down into simpler components. The resulting "secondary raw material" is used by the cell as building material and/or fuel. The phenomenon of autophagy is difficult to study and was largely unknown until the early 1990s, when Yoshinori Ohsumi conducted an impressive series of experiments with baker's yeast. He successfully identified genes required for autophagy and elucidated the mechanism of autophagy in yeast. He later showed that a similar process occurs in human cells (Doherty and Baehrecke 2018).

The technical problem for Ohsumi was the small size of the yeast cells. Since the details of their internal structure are difficult to observe under a microscope, the scientist was not sure whether yeast autophagy occurs at all. He decided to interrupt the degradation process in the vacuole, believing that this would result in the accumulation of full cellular waste autophagosomes inside the vacuole (Doherty and Baehrecke 2018). To do this, he grew mutant yeast strains that lacked the enzymes needed to break down metabolic products. At the same time, he stimulated the autophagy process by starving the yeast cells. Indeed, after just a few hours, vacuoles filled with raw waste became clearly visible under the microscope. With this method, Ohsumi confirmed the presence of autophagy in yeast cells. More important, however, was the development of methods to identify and characterize genes important in this process. By knocking out random genes with appropriate chemicals, he was able to find genes responsible for the formation of autophagosomes (Li et al. 2016).

Subsequent experiments with thousands of mutant yeast strains have identified 15 key genes involved in the autophagy process and characterized the function of the proteins they encode. It turns out that autophagy is controlled by a cascade of proteins and protein complexes, each of which controls a specific step in autophagosome formation. The announcement of Ohsumi's research in 1992 was groundbreaking. Autophagy protects cells from death by several mechanisms. One is the provision of nutrients that reduce the negative effects of endoplasmic reticulum stress and remove damaged mitochondria (Li et al. 2016).

In yeast cells, the vacuole, among other things, is responsible for the process of autophagy. During macroautophagy, a mass of cytoplasm or specific structures is sequestered in autophagosomes, which then fuse with the vacuole, releasing an internal autophagosome vesicle into the vacuole lumen, called an autophagic body (Suzuki 2012). During microautophagy, molecules destined for degradation are recruited near the membrane of the vacuole. The indentation of this membrane, followed by dissection, allows the molecules to be transported into the vacuolar lumen. In most cases, components delivered by macroautophagy and microautophagy to the interior of the vacuole are degraded by hydrolases present in the vacuole (Reggiori and Klionsky 2013). The resulting metabolites, i.e., amino acids, sugars, and nucleotides, are then transported to the cytoplasm and are used as an energy source or building blocks for the synthesis of new molecules (Delorme-Axford et al. 2015). Autophagy can be a selective or non-selective process. Both micro- and macroautophagy involve the movement of macromolecules and organelles from the cytosol into the vacuole lumen. Thus, these components must be moved from the intracellular space (also the cytosol) to the topological equivalent of the extracellular space. Moreover, during autophagy, folded proteins, macromolecular complexes, and intact organelles must be moved across the membrane, which is an important thermodynamic barrier. Certainly, autophagy is important as a response to starvation, as cells often encounter such conditions in the surrounding environment. Organelles can be eliminated by non-selective autophagy, but can also be targeted for degradation. This type of selective autophagy of organelles can occur in response to damage or dysfunction of organelles, but can also be the result of cellular adaptation to changing nutritional conditions. Selectively, autophagy occurs with the participation of vacuole aminopeptidase I (Ape1). Ape1 is synthesized in precursor form (prApe1, or precursor aminopeptidase I) and then processed in the vacuole to its mature form (mApe1, or mature aminopeptidase I). This biosynthetic pathway, which occurs under nutrient-rich conditions, is called the cytoplasm-to-vacuole targeting (CVT) pathway. Preautophagosomal structure (PAS) mediates CVT vesicle/autophagosome membrane biogenesis. In contrast, Ape1 maturation under both nutrient-rich and nutrient-poor conditions is mediated by the Atg11 protein, through its role in PAS organization. The CVT pathway is responsible for transporting the CVT complex under nutrient-rich conditions. Similarly, under starvation conditions, autophagy facilitates the transport of the CVT complex. prApe1 is the main component of the CVT complex, which is then converted into the Ape1 complex. In addition to Ape1, the CVT complex contains vacuolar α-mannosidase (Ams1) and virus-like particles Ty1 (VLP), which are produced by retrotransposons. In addition, the presence of Atg19 and Atg34 receptors is required for the CVT complex to function properly (Suzuki 2012; Reggiori and Klionsky 2013; Delorme-Axford et al. 2015).

Anhydrobiosis-induced damage to cell membranes can be repaired with the involvement of lipid droplets (LD). Intracellular lipid droplets often fuse, coming into contact with vacuole membranes and entering the vacuole (Wang 2016). Autophagy of lipid droplets (lipophagy) may be an alternative pathway for mobilizing lipids stored during metabolism after dehydration to repair damaged structures and produce energy. In the resting state of yeast cells, lipid droplets are gradually captured by vacuole membranes and are accumulated in the vacuole lumen. Similar processes occur during the dehydration and rehydration of yeast cells (Ventina et al. 1986).

Autophagy is not only a programmed process of cell death but also a chance for survival under unfavorable conditions. Just as starved organisms derive nutrients from adipose tissue resources, cells in crisis obtain energy contained in the "minor" components of the cytoplasm and require a large energy input, which limits the process. Autophagy allows cells to survive for weeks. The same cells survived for up to 2 days after autophagy was blocked. Material digested in autophagosomes provides a source of energy, nutrients, and amino acids for protein synthesis. Thus, under starvation conditions, autophagy significantly prolongs cell viability by supplying missing components (Paul and Münz 2016).

Protein products from autophagy-related genes (ATGs) play a key role in the autophagy process. At the beginning of the process, the ULK1/2-ATG13-FIP200 complex is activated, and other ATG proteins are recruited to the induction region. Two transmembrane proteins, ATG9 and vacuole membrane protein 1 (VMP1), also play an important role in the nucleation stage (Paul and Münz 2016; Wang et al. 2017).

The process of nucleation requires the activity of the class III phosphatidylinositol-3-kinase (PI3K) Vps34, the protein that regulates its activity—Vps15—and a complex composed of Barcor/mATG14 and beclin-1. Its formation requires interaction with the UVRAG protein (ultraviolet radiation resistance-related gene). This complex generates the phosphatidylinositol3,4,5-triphosphate phagophore, which is required for phagophore elongation. In addition, ATG, along with other proteins, recruits two protein-binding systems that play a key role in autophagosome formation, as well as ubiquitination systems: ATG12-ATG5-ATG16 and LC3-phosphatidylethanolamine (Paul and Münz 2016; Wang et al. 2017).

Binding processes in these systems include the protease ATG4 (which cleaves LC3 at the C-terminus), the protein ATG7, which acts as an E1-type enzyme (in both systems), and E2, ATG10 (ATG12-binding system) and ATG3 (LC3 system). The cytoplasmic material is sequestered inward as a result of the elongation of the follicular membrane. SNARE proteins are responsible for the fusion of autophagosomes and lysosomes. The inner membrane of the follicle and its contents are digested by lysosomal enzymes. Degradation results in simple molecules, mainly amino acids, which are released into the cytoplasm (Wang et al. 2017).

Under physiological conditions, basal levels of autophagy ensure that cells maintain homeostasis by participating in the turnover of proteins, organelles, and cytoplasmic components. In situations of cellular stress, autophagy increases. In addition to nutritional deficiencies, factors inducing autophagy include free radical accumulation, elevated [Ca^2+^], ER stress, and hypoxia (Minami et al. 2023).

Induction of autophagy depends on the availability of amino acids, insulin, and other growth factors, and nutrient availability as determined by AMP-activated kinase (AMPK) activity. All of these interact through the mTOR (mammalian target of rapamycin) kinase pathway, which promotes cell proliferation and protein synthesis and inhibits autophagy when nutrients are abundant. Activated mTOR phosphorylates and inactivates ULK1/2 and ATG13, preventing the induction of autophagy (Paul and Münz 2016).

The hamartin-tuberin complex (TSC1/TSC2) and the small GTP-binding protein Rheb are involved in mTOR regulation. The TSC1/TSC2 dimer triggers GTP hydrolysis, thereby inactivating Rheb and preventing mTOR activation. Numerous signaling molecules affect mTOR activity by influencing these regulators. Insulin and other growth factors activate protein kinase B (PKNB), extracellular signal-regulated kinases (ERK), and ribosomal s6 kinase (RSK) through the PI3K pathway. These kinases phosphorylate TSC2, thereby preventing the formation of TSC1/TSC2 dimers. In contrast, active AMPK kinase stimulates the autophagic process by inhibiting mTOR-dependent signaling. An increase in the AMP to ATP ratio activates AMPK through liver kinase B1 (LKB1) (Paul and Münz 2016).

The other major kinase activated by AMPK is calcium/calmodulin-dependent protein kinase (CaMKK), which is induced by high levels of Ca^2+^. Activated AMPK enhances autophagy by inhibiting the mTORC1 complex through the phosphorylation of TSC2. AMPK can also promote autophagy through the direct activation of ULK1 (Wang et al. 2017).

The mechanism of autophagy induced by endoplasmic reticulum stress involves two signaling pathways:

I. PERK (protein kinase located in the endoplasmic reticulum)/eIF2A (eukaryotic translation initiation factor 2A),
II. ERN1 (ER signal transducing protein to nucleus 1)/TRAF2 (TNF receptor-related factor 2)/JNK (N-terminal Jun protein phosphorylating kinase) (Minami et al. 2023).

In addition, Ca^2+^ release from the ER can lead to the induction of autophagy in the CaMKK/AMPK/mTORC1 pathway. Excessive accumulation of reactive oxygen species also promotes autophagy. Regulation of autophagy also involves the BCL-2 family of proteins, which regulate the activity of beclin-1. Anti-apoptotic proteins belonging to the BCL-2 family, i.e., BCL-2, BCL-XL, and MCL-1, also act as negative regulators of autophagy. Beklin-1 binds to these proteins through their BH3 domain and prevents their involvement in the initiation of autophagy. BCL-2, BCL-XL, and MCL-1 anchored to the ER membrane inhibit autophagy more effectively than those located in mitochondria. The cell location-dependent ability of the BCL-2 protein to bind beclin-1 may be related to the action of NAF-1 (nutrient deprivation autophagy factor). This protein can stabilize the BCL-2-beclin-1 complex in the ER. Loss of NAF-1 function disrupts BCL-2-beclin-1 interaction and induces autophagy (Orzechowski 2017; Holm et al. 2022).

Under conditions that stimulate autophagy, such as during starvation, some BH3-only proteins (BAD, BNIP3, NIX, NOXA, and PUMA) competitively bind the anti-apoptotic BCL2 protein baclin-1. Baclin-1 is released, allowing the formation of the PI3K complex required for phagophore nucleation. Like other pure BH3 proteins, BAX can interfere with BCL-2-beclin-1 binding but appears to inhibit rather than stimulate autophagy. This is likely related to BAX's ability to promote the degradation of beclin-1, not the BCL-2-beclin-1 complex (Orzechowski 2017; Holm et al. 2022).

In 2014, Lindquist et al. showed that the BH3 mimetic ABT-737 was unable to induce autophagy in cells lacking BAK and BAX. An alternative mechanism proposed by the authors is that BCL-2, BCL-XL, and MCL-1 proteins indirectly affect BAK and BAX, instead of inhibiting autophagy by binding to beclin-1. On the other hand, autophagy is induced by activating apoptosis through mechanisms that have yet to be elucidated. However, these results were challenged by another team, which showed that ABT-737 administration increased autophagy in cells lacking BAK1 and BAX (Humbert et al. 2020).

BCL-2 can also regulate autophagy by interacting with other proteins important for this process. In response to autophagy-inducing stimuli, ULK1 phosphorylates an activating molecule for autophagy regulated by beclin-1 (AMBRA1) and translocates this protein to the ER, where it binds to beclin-1 to form an autophagy-initiating complex (Humbert et al. 2020).

BCL-2 is present on the mitochondrial membrane and binds to AMBRA1. During autophagy, this interaction is attenuated. This indicates that BCL-2 not only inhibits autophagy by directly interacting with beclin-1, but also indirectly regulates the formation of the autophagy initiation complex. BH3-only proteins also regulate autophagy through other mechanisms. For example, NIX stimulates mitophagy by interacting with GABA receptor-associated protein (GABARAP), a functional homolog of LC3. In contrast, BIM interacts directly with beclin-1 by binding to dynein light chain 1 (DLC1) and inhibiting its action (Qin et al. 2022).

Endoplasmic reticulum stress can induce internal apoptotic pathways but is primarily a potent inducer of autophagy. Autophagy is triggered as part of the uncoiled protein response (UPR), thereby removing misfolded proteins and their aggregates. In addition, the damaged membrane of the endoplasmic reticulum is degraded during reticulum phagocytosis. Thus, autophagy helps maintain endoplasmic reticulum function and protects cells from apoptosis (Wang et al. 2022).

The cytoprotective role of autophagy has been confirmed in various experimental models, showing that inhibition of autophagy under endoplasmic reticulum stress significantly promotes apoptosis. More recently, studies have demonstrated that activation of autophagy precedes apoptosis induced by endoplasmic reticulum stress. Moreover, the sequence of events is independent of the type of stimulus, and the intensity of autophagy determines the threshold for apoptosis induction (Wang et al. 2022).

Another mechanism by which autophagy prevents cell death is the removal of damaged mitochondria. Increased permeability of the inner mitochondrial membrane induced by intracellular stress signals leads to loss of mitochondrial membrane potential and mitochondrial outer membrane permeabilization (MOMP). Damaged mitochondria are removed by mitophagy. However, if depolarization affects the majority of mitochondria, and cells do not remove mitochondria by autophagy, apoptosis begins. Thus, mitophagy directly raises the threshold for activation of the intrinsic apoptosis pathway, opposing MOMP. Moreover, depolarized mitochondria are a source of free radicals and thus indirectly protect cells from death (Kasprowska-Liśkiewicz 2017).

Excessive degradation of cell contents can lead to cell death by autolysis. This concept and numerous observations of autophagy activation under stress leading to cell death have given rise to the term autophagic cell death (ACD). This term refers to a form of death other than apoptosis and necrosis, the main characteristic of which is the presence of numerous vesicles with a double membrane containing cytoplasmic fragments (Kasprowska-Liśkiewicz 2017). ACD occurs in at least two situations. ACD is involved in the removal of specific cells during *Drosophila melanogaster* development and in the death of apoptosis-incompetent tumor cells in response to specific chemotherapeutic agents in vitro (Kasprowska-Liśkiewicz 2017).

There is a growing view that autophagy is a cytoprotective mechanism and that cell death accompanies rather than results from autophagy. However, the question of autophagy's involvement in carrying out cell death appears to remain unresolved. Recently, a mechanism of cell death that relies on the autophagic machinery but has morphological features distinct from ACD has been identified. This unknown mechanism has been named "autosis" (*autophagy*) (Yamamoto et al. 2016). Activation of autosis can be triggered by starvation, some forms of ischemia, or a massive increase in autophagy induced by the administration of autophagy-inducing peptides. A unique feature of autophagy is its dependence on Na^+^/K^+^ ATPase and the development of chromatin condensation, which is not observed in ACD (Yamamoto et al. 2016).

## Conclusion

The present review work was aimed at introducing and explaining the essence of such a complex process as anhydrobiosis. The phenomenon under discussion involves a wide group of both unicellular and multicellular organisms. Although the phenomenon of anhydrobiosis in the research most often concerns microorganisms, namely yeast, it should be remembered that some species of insects and plants have acquired the ability to survive in unfavorable conditions thanks to this process. With the high degree of urbanization, increased human interference with nature, and the observed climate changes, the group of organisms for which environmental conditions are becoming increasingly unsuitable is increasing. Therefore, there is a possibility that the use of anhydrobiosis will be acquired over time by more organisms.

Anhydrobiosis is a state of living organisms during which their metabolism is suspended or delayed due to a high degree of dehydration. It is a reversible process, but transition into this state can involve the loss or damage of valuable cellular components, and in critical cases can lead to death. The topic of anhydrobiosis gained popularity at the turn of the twentieth century when scientists tried to explain the process. Unfortunately, researchers were limited by the experimental methodologies and existing laboratory techniques of that time and, despite interesting hypotheses, were often unable to confirm them in their research. With the development of modern laboratory techniques and methods, the topic of anhydrobiosis is gaining prominence in the literature, and modern research is providing answers to questions raised more than 50 years ago.

Yeasts are one of the most important groups of microorganisms, used extensively in food technology and pharmacology, and are also involved in attempts to manage waste from various industries. They are widely used now as the optimum model of any eukaryotic cell. For this reason, they have also become model microorganisms for the study of anhydrobiosis. The complex set of factors that determine the correct and most efficient course of anhydrobiosis includes, among others, the initial state of yeast cells, the state of the cell wall and membrane, the content of storage sugars (trehalose, glycogen) and the enzymes responsible for their metabolism, as well as the level of lipid droplets or antioxidant mechanisms headed by glutathione. As described in this review, these factors play an important role in the course of anhydrobiosis, and it is worth noting that each of the compounds has its own specific functions and probably is irreplaceable.

Anhydrobiosis offers a potential avenue for innovative cell preservation techniques, particularly pertinent in the context of transplantation, where maintaining organs in an anhydrobiotic state could enhance their durability and availability for awaiting patients. Anhydrobiosis further holds promise in streamlining the production of stable vaccines, facilitating their storage and distribution in hard-to-reach locales.

Looking ahead, anhydrobiosis bears significance in agriculture and environmental conservation. Storing plant seeds in an anhydrobiotic state holds the potential to contribute to the preservation of biological diversity and the safeguarding food resources amidst shifting climatic patterns. Leveraging anhydrobiotic mechanisms could also expedite the development of more efficacious bioremediation methodologies, enabling microorganisms to endure adverse environmental conditions and ameliorating the processes of decontaminating polluted regions.

It is important to continue research towards a more thorough understanding of the mechanisms of anhydrobiosis, as well as subjecting various microorganisms to this process because, despite the progress to date, literature gaps still remain in the topic under discussion.
